# Comparison of Scoring Systems in Predicting Success of Percutaneous Nephrolithotomy

**DOI:** 10.4274/balkanmedj.2017.1631

**Published:** 2019-01-01

**Authors:** Muzaffer Akçay, Muhammed Tosun, Fatih Gevher, Senad Kalkan, Cevper Ersöz, Yunus Kayalı, Abdulkadir Tepeler

**Affiliations:** 1 Department of Urology, Bezmialem Vakıf University School of Medicine, İstanbul, Turkey; 2 Clinic of Urology, Private Sen Jorj Avusturya Hospital, İstanbul, Turkey

**Keywords:** Percutaneous nephrolitotomy, scoring methods, specificity and sensitivity, urinary calculi

## Abstract

**Background:**

Scoring systems are useful to inform the patients about the success and complication rates of the operation prior the surgery.

**Aims:**

To determine the applicability of the popular scoring systems (Guy’s, stone size, tract length, obstruction, number of involved calices, and essence/stone density and Clinical Research Office of the Endourological Society) by means of examining preoperative data of patients treated with percutaneous nephrolithotomy.

**Study Design:**

Cross sectional study.

**Methods:**

We retrospectively reviewed files of the patients who had undergone percutaneous nephrolithotomy in our center between 2011 and 2015. Excluded from the study were patients aged <18 years, and those who were not assessed preoperatively with computed tomography. Preoperative computed tomography images of all patients were assessed by a single observer, and patients were graded based on three scoring system. Demographic data were analyzed along with perioperative data (operation, fluoroscopy, length of hospital stay, changes in hematocrit values, location, and number of access sites, stone-free and complication rates).

**Results:**

A total of 298 patients who had been treated with 300 procedures were enrolled into the study. Mean age, stone burden, number of stones, and density were 48.1±12.9 years, 663.5±442.8 mm^2^, 1.8±1.1 and 888.3±273 HU respectively. Scores of the cases based on Guy’s, stone size, tract length, obstruction, number of involved calices, and essence/stone density, and Clinical Research Office of the Endourological Society scoring system were calculated as 2, 7.6, and 222.1 points respectively. 81.6% of the patients were stone-free. Complications were detected in 30 (9.9%) patients. Based on receiver operating characteristic curve analysis a positive correlation was detected between success rate and scoring systems, i.e., Guy’s (p=<0.001, r=-0.309), stone size, tract length, obstruction, number of involved calices, and essence/stone density (p=<0.001, r=-0.295), and Clinical Research Office of the Endourological Society (p=<0.001, r=0.426). The Clinical Research Office of the Endourological Society scoring system had the highest predictive value. The sensitivity rates rates for Guy’s, Clinical Research Office of the Endourological Society and Stone scoring system were as 78.78%, 80% and 82.34% respectively.

**Conclusion:**

All of scoring systems predicted correctly the success of the percutaneous nephrolithotomy procedures. The Clinical Research Office of the Endourological Society scoring system had the highest predictive value.

Urinary system stones are among the most common disease throughout the world and are regarded as a serious health problem. During the last 30 years, their incidence and prevalence have increased greatly throughout the world. In the 1970s, the prevalence rate was 3.2%, rising to 8.8% in 2010 (1,2). According to one study, there was a 10% increase in hospitalization related to renal stones (3). Alongside this increase, efforts and tools used to treat urinary system stones have advanced tremendously. Minimally invasive methods, such as shock wave lithotripsy (SWL), retrograde intrarenal surgery, and percutaneous nephrolithotomy (PNL), have been developed. PNL is especially recommended as a first-line treatment for renal stones larger than 2 cm, and the number of PNL procedures has risen as a result of the increased incidence of stone disease (4,5,6).

In recent years, scoring systems (SS) that predict success rates before the operation have been developed. These SS are useful to inform the patients about the success and complication rates of the operation prior to surgery (7). These SS are based on the parameters related to the stone (size, location, and density), the patient (anatomical anomaly and previous treatments), and the surgeon (experience).

The Guy’s, Clinical Research Office of the Endourological Society (CROES), and stone size, tract length, obstruction, number of involved calices, and essence/stone density (S.T.O.N.E.) SS are the most often used SS that have been introduced into clinical practice (8,9,10). In our study, we aimed to compare the predicted success rates between these three SS by means of examining preoperative data on patients who had undergone PNL in our clinic. To the best of our knowledge, this is the first clinical study comparing three SS using patient data from a single center.

## MATERIALS AND METHODS

After obtaining approval from the ethics committee, we retrospectively reviewed the files of patients who had undergone PNL between November 2011 and September 2015 in our clinic. A total of 298 patients were treated during this period. Two patients had PNL operation on each kidney. The total number of PNL operations performed was 300. Informed consent was obtained from all patients. All the operations were performed by three different surgeons. Patients aged <16 years and those who were not assessed preoperatively with computed tomography (CT) were excluded from the study. Patients who had undergone secondary procedures to achieve complete stone-free status were not enrolled. Demographic data [age, gender, body mass index (BMI), previous renal stone treatment, stone burden, location, and number of stones] and perioperative data (operation, fluoroscopy, length of hospital stay, changes in hematocrit values, location and number of access sites, and stone-free and complication rates) were analyzed.

Preoperative and postoperative non-contrast CT images of all patients were assessed by a single observer (MT), and patients were graded on the basis of three SS. In the Guy’s system, the number and location of stones, presence of staghorn stone, anatomical abnormality, and spinal cord injury or spina bifida (if any) were classified as grades 1, 2, 3, or 4 (8). In the S.T.O.N.E. SS, the stone burden and density (HU), length of the tract (skin to stone), presence of obstruction and location and number of the stones were scored from 1 to 4 and classified thusly (9). In the CROES nomogram, the location and number of stone(s), previous treatment status, presence of staghorn stone (if any), and average case volume were evaluated on the basis of the diagram (10).

### Surgical technique

All procedures were performed under general anesthesia, and ureteral catheterization was performed using a 5-6 fr ureter catheter (Geotek, Ankara, Turkey) with the patients placed initially in the lithotomy position and then turned to the prone position. Retrograde pyelograms were obtained, followed by fluoroscopy-guided access into the collecting system. After a sensor guide (Sensor TM Gide Wire, Boston Scientific, USA was sent to the collecting system, the tract was dilated using sequential amplatz dilators (13-30 fr, amplatz dilatator set, Boston Scientific, USA). We then entered the collecting system using a nephroscope (12-30 fr, Nephroscope, Karl Storz, Germany). The stones were fragmented using an ultrasonic, pneumatic lithotripter or Ho:YAG laser. If necessary, an additional tract was created to reach any remaining calculi. At the end of the procedure, a nephrostomy tube was, at the preference of the surgeon, placed in cases of collecting system perforation, bleeding, or residual stones.

On the postoperative first day, the patients were evaluated with laboratory tests and plain graphy of the kidneys, ureters, and bladder (KUB). After removal of their nephrostomy tubes, the patients were discharged on postoperative days 1-3. Postoperative complications were evaluated on the basis of the Clavien system (11). At the postoperative first month control visit, stone-free rates were evaluated with non-contrast CT.

### Statistical analysis

Data were collected using IBM SPSS version 22. Continuous variables were compared using the independent sample t-test, and the results are presented as means and standard errors of the means. Categorical variables were compared using Fisher’s exact or chi-square tests, and the outcomes are presented as percentages. Correlation analyses were performed using the Pearson correlation coefficient (r). Two-tailed p values <0.05 were considered statistically significant. Receiver operating characteristic (ROC) curves were generated for each SS. The area under the curve and asymptotic 95% confidence interval were calculated for each ROC curve. ROC curves were drawn to evaluate the accuracies of the SS for pre-operative prediction of the success rate. Power analysis revealed that, in order for an effect of this size to be detected (80% chance) as significant at the 5% level, a sample of 234 participants would be required.

## RESULTS

A total of 298 patients who underwent 300 PNL (205 male, 95 female) procedures were enrolled in the study. The patient’s mean age and BMI were 48.1±12.9 (16-83) years and 28.3±4.4 (18-52.4) kg/m^2^, respectively. The mean stone burden, number, and density were 663.5±442.8 (96-2826) mm^2^, 1.8±1.1 (1-6), and 888.3±273 (409-1605) HU, respectively. Staghorn stones were detected in 33 patients. The patients’ mean Guy’s, S.T.O.N.E, and CROES scores were calculated as 2, 7.6, and 222.1 points, respectively (Table 1).

The mean procedure time, fluoroscopy time, and length of hospital stay were 69.7±33.8 (20-240) minutes, 124.6±96.5 (28-660) seconds, and 2.2±1.05 (1-9) days, respectively. The mean decrease in hematocrit was 3.8±2.7% (0.8%-15.6%). The mean number of percutaneous renal tracts was 1.1±0.3 (1-3) (Table 1). Stone-free status was determined in 245 (81.6%) patients on the basis of control CTs. Postoperative complications were detected in 29 (9.9%) patients. These were fever (n=5; Clavien grade 1), urinary system infection (n=3; Clavien grade 2), colicky pain which regressed with medical treatment (n=3; Clavien grade 1), prolonged urine leakage requiring ureteral stent insertion (n=9; Clavien grade 3a), bleeding requiring transfusion (n=5; Clavien grade 2) or angio-embolization (n=1; Clavien grade 3b), extravasation requiring placement of a drainage catheter (n=1; Clavien grade 3a), pneumothorax necessitating implantation of a chest tube (n=1; Clavien grade 3a), and urosepsis (n=1; Clavien grade 4).

Between cases which were stone free and those which were not, statistically significant differences appeared in terms of stone size (p<0.001), location (p<0.001), procedure time (p<0.001), length of hospital stay (p<0.001), number of access tracts (p=0.021), complication rates (p<0.001), and Guy’s (p<0.001), CROES (p<0.001), and S.T.O.N.E. (p<0.001) scores.

**Figure 1:**
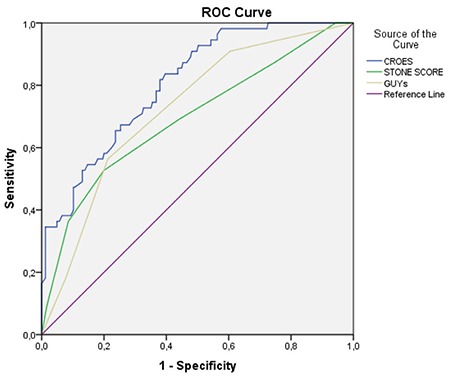
Area under ROC curves as diagnostic ability of the each tests. CROES: Clinical Research Office of the Endourological Society; ROC: receiver operating characteristic; S.T.O.N.E.: stone size, tract length, obstruction, number of involved calices, and essence/stone density

Figure 1 and Table 2 show the evaluation of SS based on ROC curve analysis. A positive correlation was detected between PNL-related success rates and the three SS, i.e., Guy’s (p<0.001, r=−0.309), S.T.O.N.E. (p<0.001, r=−0.295), and CROES (p<0.001, r=0.426). The CROES SS had the highest predictive value, specificity, and sensitivity.

## DISCUSSION

Accurately predicting success and complication rates after a PNL procedure may be difficult, even in referral centers with high case volumes. Outcomes of the PNL procedures are related to factors of the stone (stone burden, density, and location), the patient (obesity, anatomical anomaly, previous treatment), and the surgeon (experience) (8,9,10,11). The idea of predicting procedural outcomes by means of evaluation of these factors stimulated the development of SS. Using these SS, prior to surgery, patients can be provided with more precise information about the complication and success rates of an operation. Different SS have been improved to anticipate postoperative outcomes by taking into consideration factors that could influence the procedure result (12,13,14).

The Guy’s SS is based on abdominal radiograms. It considers the location and number of stones, staghorn status, and anatomic abnormalities to construct four grades (8). The Guy’s SS is simple and easily applicable compared with the CROES and S.T.O.N.E. systems. Ingimarsson et al. (15) retrospectively evaluated 166 patients treated with PNL and reported that the Guy’s SS was successful in predicting success rates and postoperative complications. Vicentini et al. (16) concluded that the Guy’s SS predicts operative time, blood transfusion, and complication rates in addition to success rates.

The S.T.O.N.E. SS was first introduced by Okhunov et al. (13) It is based on the evaluation of preoperative CT according to five main criteria: stone size, length of access tract, obstruction, number of calculus calices, and stone density (9). Different from the CROES and the Guy’s SS, S.T.O.N.E. SS evaluates the parameters of stone density, grade of obstruction, and length of the access tract. In their multicentric retrospective study, Okhunov et al. (17) detected correlations between the S.T.O.N.E. system and success and complication rates, blood loss, operative time, and length of hospital stay.

Smith et al. (14) also described a nomogram using radiological (location, number, and size of the stone(s), and presence of staghorn stone), and clinical parameters (case volume per year and prior treatment) and called it the CROES SS (10). The CROES SS is different from the other two SS because it uses clinical parameters such as case volume per year and prior treatment; it also has a positive correlation between total score and success rates. Sfoungaristos et al. (18) found that the CROES system successfully predicted postoperative outcomes. They also reported that the stone number and number of calculus calices affected the success rate. We also believe that higher numbers of stones and calculus calices yields higher access numbers. In the present study, the number of accesses performed was higher in the non-stone-free group, which confirms that higher access numbers are related to less favorable results.

Bozkurt et al. (19) compared the Guy’s and the CROES SS on 437 patients and found that both systems were correlated with procedural success. Their ROC curve analyses showed the CROES to be more sensitive and specific. Both SS were correlated with complications, blood loss, and operative times (19). In another study, Noureldin et al. (20) compared the Guy’s and S.T.O.N.E. systems; they also showed that both systems were correlated with blood loss, operative time, and length of hospital stay, in addition to postoperative success.

From the literature, we found three studies that examined the predictive capacity of these three SS (21,22,23). One of them, published by Labadie et al. (21) showed accurate prediction of postoperative success rates. According to their study, the most valuable results were obtained using the CROES SS. The Guy’s and S.T.O.N.E SS also correlated with blood loss and length of hospital stay; however, certain patients with some parameters used in the CROES SS (e.g., history of SWL, URS, and open surgery) were excluded from the study (21). In another multicentric study, Tailly et al. (22) detected that one of every three SS were related to procedural success. Finally, Sfoungaristos et al. (23) compared the Guy’s, CROES, and S.T.O.N.E. SS for staghorn stones; they found that all three SS were significantly predictive of stone-free status, but in multivariate analyses, only the S.T.O.N.E. SS was superior to the other two in the prediction of procedural success (23).

Our study has some limitations. It is retrospective in nature, and the patient numbers may be considered relatively low. Also, the procedures were carried out by three different surgeons. Although the surgeons who performed the operations have vast experience in endourological procedures, this might have affected the overall stone-free status. In order to minimize the influence of this situation on the evaluation, all pre- and postoperative non-contrast CT images were assessed by a single observer (MT). Nonetheless, our study has merit and deserves consideration because, in contrast to other published studies, all three SS were evaluated using data from patients at the same referral center.

In conclusion, the CROES SS had the highest predictive value, which we attribute to evaluation of radiological findings, yet the results showed that all of them predicted the success of the PNL procedures. These three SS should experience more widespread use. Further large-scale prospective studies would help to support the positive outcomes reported thus far.

## Figures and Tables

**Table 1:**
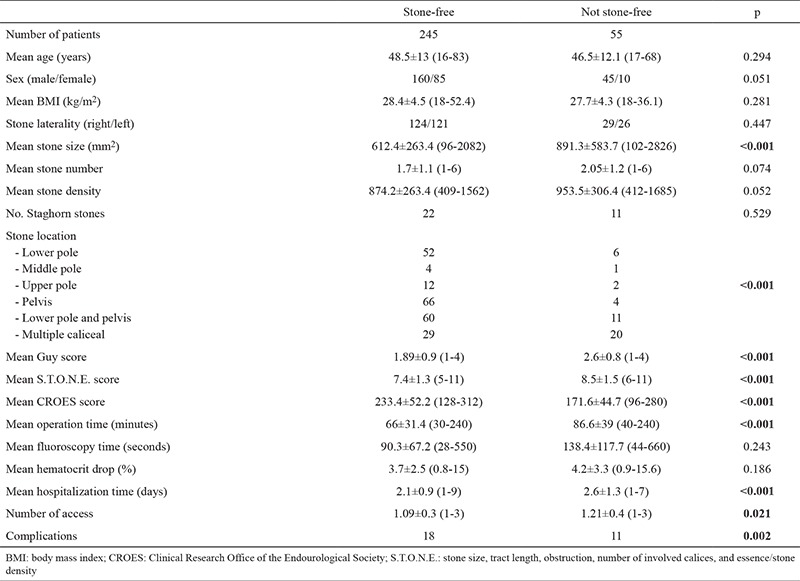
Demographics, preoperative scores, and outcomes

**Table 2:**
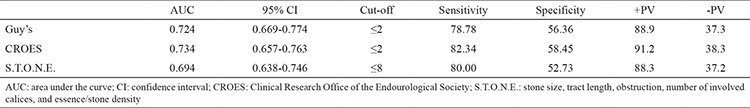
Receiver operating characteristic curve analysis for each scoring systems
